# Survey of water proton longitudinal relaxation in liver in vivo

**DOI:** 10.1007/s10334-021-00928-x

**Published:** 2021-05-12

**Authors:** John Charles Waterton

**Affiliations:** 1 Centre for Imaging Sciences, Division of Informatics Imaging and Data Sciences, School of Health Sciences, Faculty of Biology Medicine and Health, University of Manchester, Manchester Academic Health Sciences Centre, Oxford Road, Manchester, M13 9PL UK; 2 Bioxydyn Ltd, Rutherford House, Manchester Science Park, Pencroft Way, Manchester, M15 6SZ UK

**Keywords:** Liver, Magnetic resonance imaging, Biomarker, *T*_1_ relaxation time, Reproducibility

## Abstract

**Objective:**

To determine the variability, and preferred values, for normal liver longitudinal water proton relaxation rate *R*_1_ in the published literature.

**Methods:**

Values of mean *R*_1_ and between-subject variance were obtained from literature searching. Weighted means were fitted to a heuristic and to a model.

**Results:**

After exclusions, 116 publications (143 studies) remained, representing apparently normal liver in 3392 humans, 99 mice and 249 rats. Seventeen field strengths were included between 0.04 T and 9.4 T. Older studies tended to report higher between-subject coefficients of variation (CoV), but for studies published since 1992, the median between-subject CoV was 7.4%, and in half of those studies, measured *R*_1_ deviated from model by 8.0% or less.

**Discussion:**

The within-study between-subject CoV incorporates repeatability error and true between-subject variation. Between-study variation also incorporates between-population variation, together with bias from interactions between methodology and physiology. While quantitative relaxometry ultimately requires validation with phantoms and analysis of propagation of errors, this survey allows investigators to compare their own *R*_1_ and variability values with the range of existing literature.

**Supplementary Information:**

The online version contains supplementary material available at 10.1007/s10334-021-00928-x.

## Introduction

The liver longitudinal water proton relaxation rate *R*_1_ is important for several reasons. Native *R*_1_ is a biomarker of liver pathology [1, 2]. Also, other liver biomarkers are secondarily derived from *R*_1_ measurements: for example, increase in *R*_1_ post-gadoxetate is a biomarker of hepatocyte function [3, 4]; extracellular volume is derived by comparing *R*_1_ pre and post contrast [5]; and baseline *R*_1_ is required for rate constants in dynamic contrast-enhanced MR [6], for tissue oxygen tension in oxygen-enhanced MR [7], and for relaxivity measurements in contrast agent research[8].

Measurements of *R*_1_ in individual livers or liver regions suffer from both systematic errors and random errors [9]. Systematic errors (bias) arise because measurements are imperfectly performed. Other systematic deviations occur because different methods, even when perfectly performed, yield *R*_1_ values with different dependences on liver composition and physiology. Random (repeatability) errors arise from physiologic and instrument noise, and can be high particularly when regions-of-interest are small. In addition, even in the absence of bias and noise, there are, in each study, genuine between-subject differences in *R*_1_ due to between-subject variation in physiology or subclinical pathology.

To mitigate the effects of random error in establishing a “normal” or “baseline” liver *R*_1_, investigators sometimes employ a "compromise" *R*_1_, averaged from all subjects in their study. This likely reduces the "noise" variance, but introduces other errors by ignoring true between-subject variation. Other investigators may obtain *R*_1_ from literature reports, although this will introduce additional bias if different measurement methods had been used, or different populations had been studied.

The aim of this study was to survey values, and variabilities, of normal liver *R*_1_ from the published literature. This would give investigators an indication of whether the liver *R*_1_ or *T*_1_ values and variabilities they measure are broadly consistent with, or discordant from, the prior literature.

## Methods

### Literature searching

Literature was searched manually using "Ovid Medline" (www.ovid.com) for “magnetic resonance imaging” AND “liver” AND “relaxation”. Additional literature reports were retrieved from citations, supplemented by a more intensive search for data with B_0_ = 4.7 T, 7 T, 9.4 T, 11.7 T, 14.1 T or 21.1 T (see supplementary material 1 for further details). Liberal inclusion criteria were employed: any report, in any language, which claimed to measure liver *R*_1_ or *T*_1_ was included, irrespective of methodology or study design. Studies where B_0_ was unclear, or where liver *R*_1_ or *T*_1_ was measured but not reported, were necessarily excluded. Studies using Look-Locker methods were included if they reported *T*_1_ or *R*_1_, but excluded if they reported an apparent *T*_1_* only. Human and rodent subjects were included if they were normal controls of any age, if the study reported normal parts of livers with focal disease, or if they were patients in whom no liver abnormality had been found. Studies of definitely pathological liver, suspected duplicates, and ex vivo studies were excluded.

### Analysis

The mean and variance of *R*_1_ across all subjects in each study was estimated from the publications, with the coefficient of variation given by ${\text{CoV}}=\sqrt{\text{variance}}/{\text{mean}}$. Where measurements were made on the same subjects using the same method (repeatability), the weighted mean ± SD was used, however where measurements were made on the same subjects using different method (e.g., different field strengths) the measurements were treated as if from two different studies. Any *R*_1_ measurement method was allowed, as long as *T*_1_ (s) or *R*_1_ (s^−1^) was reported. Where *T*_1_ ± SD was reported, a point estimate of *R*_1_ was estimated as *T*_1_^−1^ and the between-subject variance in *R*_1_ was estimated (see supplementary material 2) as:

1 $$ 0.25\left( {\left( {\left( {T_{1} - SD} \right)^{ - 1} } \right) - \left( {\left( {T_{1} + SD} \right)^{ - 1} } \right)} \right)^{2} $$

**Fig. 1:**
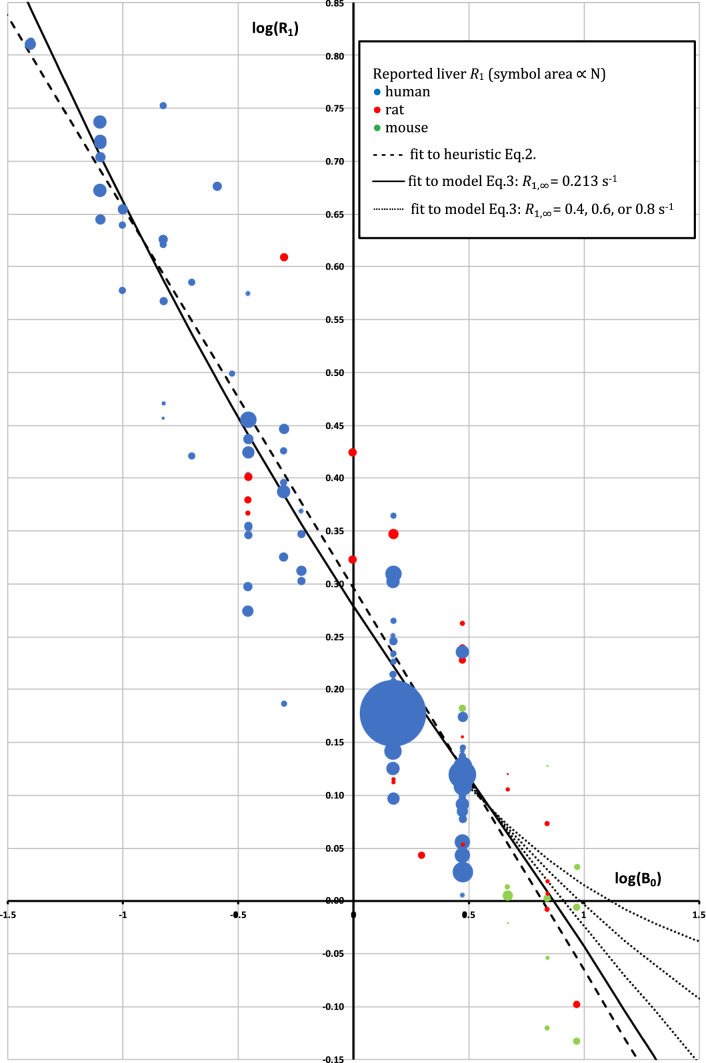
Log–log dependence of longitudinal relaxation rate on field strength. Blue: human; Red: rat; Green: mouse. Each symbol represents one study. Size of circle reflects number of subjects (some smaller symbols are occluded by larger symbols). Dashed black line: fit to Eq. 2. Solid black line: fit to Eq. 3 with ${R}_{1,\infty }$= 0.213 s^−1^. The dotted line illustrates, for the benefit of investigators working at > 10 T, fits to Eq. 3 where ${R}_{1,\infty }$ was fixed at higher values of 0.4 s^−1^, 0.6 s^−1^, and 0.8 s^−1^, intermediate between 0.213 s^−1^ and the 0.9–1.0 s^−1^ value observed at 9.4 T in Table 1

**Fig. 2:**
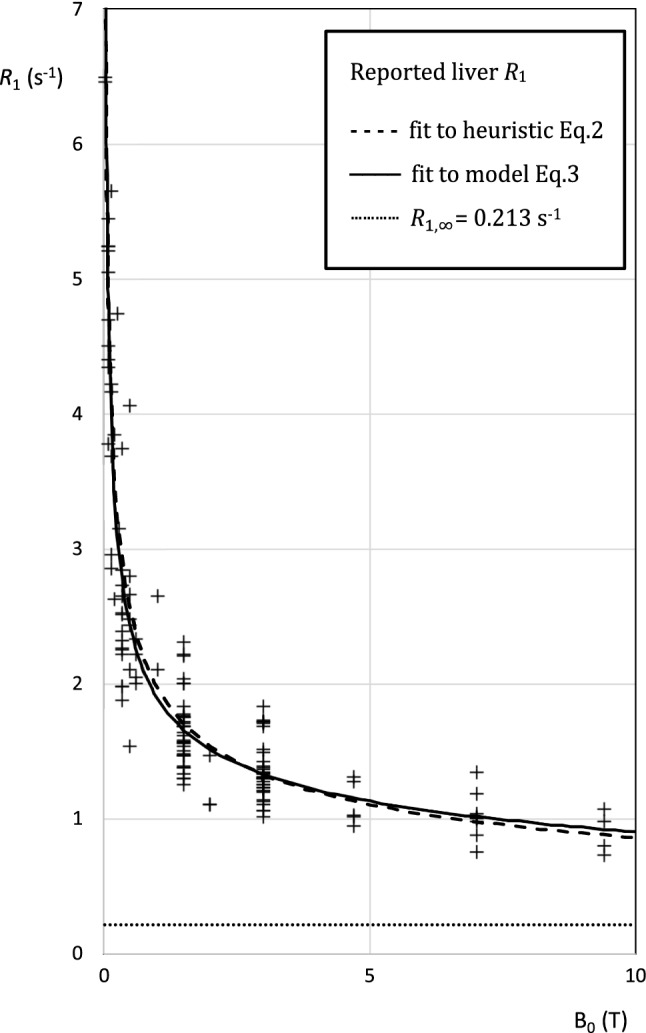
Dependence of longitudinal relaxation rate on field strength. Each symbol represents one study. Dashed black line: Eq. 2. Solid black line: Eq. 3. Dotted line: ${R}_{1,\infty }=0.213 {s}^{-1}$

In a few cases, the between-subject variance in *R*_1_ was estimated from a bar or scatterplot depicted in the publication, or from the range rule [10]. To aggregate the data, individual studies were weighted by the inverse of their between-subject variance in *R*_1_. Studies with *N* = 1, or where a variance could not be extracted, were included in Figs. 1 and 2, but their *R*_1_ was assigned zero weight in the fits. In addition, a method to account for the well-known B_0_-dependence of liver *R*_1_ [11–15] was needed. Two methods of representing this B_0_ dependence were used: a heuristic log–log relationship, and a biophysical power-law model developed by Diakova et al. [12]. *R*_1_ was fitted to B_0_ using the weighted non-linear least squares function nls() in R[16] (see supplementary material 3). The fitted parameters in the heuristic were *M* and *C*:

2 $$ \log \left( {R_{1} } \right) = M\log \left( {{\text{B}}_{0} } \right) + C $$

The fitted parameters in the model were *A* and *B*:

3 $$ R_{1} = A\omega^{k} + B\tau_{D} \left[ {\ln \left( {1 + \left( {\omega \tau_{D} } \right)^{ - 2} } \right) + 4\ln \left( {1 + \left( {2\omega \tau_{D} } \right)^{ - 2} } \right)} \right] + R_{1,\infty } $$

where ${R}_{1,\infty }$ is the high-frequency asymptote, i.e., the extreme narrowing condition, set here to 0.213 s^−1^ at 310 K[17]; ${\tau }_{D}$ is the translational correlation time from Diakova et al. [12] adjusted for temperature to 1.43 × 10^–11^ s; $k=-0.6$ also from Diakova et al. [12]; and $\omega =2\pi \times 42.58\times {10}^{6}\times {\text{B}}_{0}$ s^−1^. In the summaries, lower (LQ) and upper (UQ) quartiles, and medians, are reported. For exploratory fits using other weightings, see Supplementary Material 4.

## Results

**Fig. 3:**
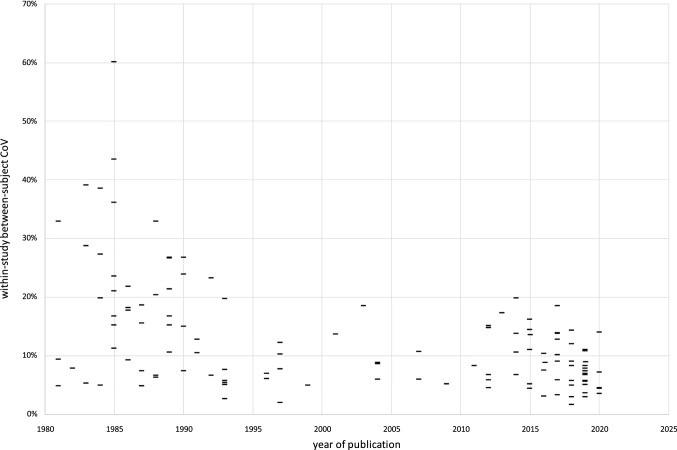
Within-study between-subject coefficient of variation as a function of year of publication

Approximately 500 publication abstracts were read, from which around 270 publications were selected and reviewed. After exclusions, 116 publications remained, with publication dates between 1981 and 2020. Some publications reported multiple studies, or multiple groups within a single study, so that 143 studies were available to contribute to this analysis. These represented 3392 humans [1–4, 7, 11, 14, 15, 18–94], 99 mice [95–105] and 249 rats [5, 33, 105–126]. The number of subjects per study varied between 1 and 1037 (median 12). A very wide variety of *T*_1_ measuring methods was used. Frequently used approaches (see supplementary material 5) were inversion-recovery (18% of studies), saturation-recovery (21%) or variable-flip-angle (10%), which compare signal arising respectively when inversion time, repetition time, or flip angle are incremented. The median number of increments was 3 (range 2–20). Various read-outs were employed including spin-echo, gradient-echo, echo-planar or localized spectroscopy. Other studies employed variants of Look-Locker (24%) or MR fingerprinting (1%). Some studies reported that they suppressed fat, and/or corrected for iron-induced *T*_1_-shortening; some reported motion suppression, registration, triggering, gating or breath-hold; some reported B_1_ correction or phantom-based validation. Some studies analysed quite small regions of interest often avoiding blood vessels and bile ducts; others included most or all of the liver. Seventeen field strengths were included between 0.04 T and 9.4 T. No values were found in reports using B_0_ > 9.4 T: one report of ${T}_{1}^{*}=1.0\pm 0.1\mathrm{ s}$ at 14.1 T was excluded[127]. Figures 1 and 2 show plots of *R*_1_ against B_0_, in which *R*_1_ shows the expected decrease with increasing field: Table 1 gives values for the most important field strengths. The fit to Eq. 2 gave $M=-0.3611\pm 0.0115$ and $C=0.2956\pm 0.0073$. The fit to Eq. 3 gave $A=(8.663\pm 0.681)\times {10}^{4}$ and $B=(1.294\pm 0.082)\times {10}^{9}$. An exploratory attempt at a three-parameter fit to Eq. 3 (i.e., to *A*, *B*, and ${R}_{1,\infty }$) failed to provide evidence for ${R}_{1,\infty }>0$ (supplementary material 4). When data were subgouped by species or by method, no evidence was found that the subgoup *R*_1_ values deviated systematically from Eq. 3 (supplementary material 6). Across all studies, the median between-subject CoV was 9.1% (LQ 5.9%, UQ 16.5%, rms 17.0%). There was, however, a tendency for early studies to report high between-subject CoV (Fig. 3 and supplementary material 7): no study published after 1992 had CoV ≥ 20%, and for post-1992 studies the median between-subject CoV was 7.4% (LQ 5.6%, UQ 11.0%, rms 9.6%). In half those studies, the measured *R*_1_ deviated from Eq. 3 by 8.0% or less (LQ 2.8%, UQ 16.6%).

**Table 1:** Preferred *R*_1_ values (s^−1^) for five commonly used field strengths, derived from the data and from the fits

B_0_ (T)	Mean over studies (N studies)	Weighted mean over studies (N studies)	Mean over subjects (N subjects)	Fitted to heuristic Eq. 2 (143 studies /3740 subjects)	Fitted to model Eq. **3**
9.4	0.90 (4)	1.01(4)	0.89(38)	0.88	**0.92**
7	1.02 (9)	1.02(9)	1.00(56)	0.98	**1.02**
4.7	1.12 (5)	1.22 (5)	1.05(34)	1.13	**1.15**
3	1.34 (36)	1.42(36)	1.29(989)	1.33	**1.33**
1.5	1.66 (37)	1.47(37)	1.55(1700)	1.71	**1.66**

Five different methods of generating a preferred *R*_1_ are illustrated: the model fit (in bold) makes greatest use of the available information

At each field strength, there was considerable variation in *R*_1_ between studies: the between-study CoV was 16% for post-1992 studies. Six publications[2, 37, 98, 119, 128, 129] also reported liver *R*_1_ repeatability (same subject, different scan, same measurement conditions): the rms CoV was 1.9%. These CoVs allowed a crude estimate (supplementary material 8) of the relative size of the three main variance components: repeatability variance contributed ~ 1%; within-study-between-subject variance contributed ~ 25%; and between-study variance contributed ~ 74%.

## Discussion

In liver, as in pure water, both intramolecular and intermolecular water ^1^H-^1^H dipolar relaxation contribute to *R*_1_. Specific additional contributors to water ^1^H *R*_1_ in liver arise from ^1^H-^1^H dipolar relaxation between water and other molecules, and ^1^H-electron dipolar relaxation between water and various iron- or copper-containing substances or dioxygen. These ^1^H-containing and unpaired-electron-containing substances differ in concentration between subjects. The liver ^1^H resonance arises mostly from tissue water in hepatocytes. Other contributions come from water in other intracellular compartments (e.g., Kupffer cells, erythrocytes), and in extracellular compartments (e.g., bile, plasma, space of Disse). Signal from triglyceride and inflowing blood may contribute, depending on the sequence used. Macromolecules contribute to the signal, notably collagen and glycogen which have different concentrations in different subjects. These factors likely account for some of the variation between subjects and between studies.

Fits from the heuristic and from the model were very similar. The main difference is that the heuristic forces *R*_1_ to zero at infinite field, while the model forces *R*_1_ to asymptote in the extreme narrowing condition. This difference might become important at fields above 7 T (Fig. 1). In this study, following Diakova et al.[12], the asymptote ${R}_{1,\infty }$ was fixed at 1/4.7 s^−1^, equal to the *R*_1_ of pure deoxygenated water at 310 K at high field [17]: a slightly higher value would be more appropriate if *R*_1_ values from liver water and pure water do not converge as illustrated in Fig. 1.

The relative magnitude of the major variance components was estimated. This is very crude, and given the heterogeneity and variable quality of the raw data, should be considered a rough guide only. The within-study between-subject CoV reflects not only repeatability error (~ 1% of the variance), but also the expected between-subject variation (~ 25% of the variance). Between-study variation (~ 74% of the variance) also includes between-population variation, together with bias from interactions between each study’s measurement method and its livers’ variation in flow, motion, fat, oedema, collagen, glycogen and iron. *R*_1_ may also change after a meal [89], during the menstrual cycle [25] or with drug treatment [25].

The literature survey was not fully PRISMA-compliant [130] and is unlikely to be complete. Studies explicitly of liver *R*_1_ or *T*_1_ as a biomarker are readily retrieved, because appropriate keywords are generally used in the title and abstract. However, for studies where liver *R*_1_ or *T*_1_ measurement is incidental to another objective, for example extracellular volume, relaxivity, or dynamic contrast-enhanced studies, suitable keywords may not have been included.

There is no single “correct” value for any liver’s ^1^H *R*_1_. *R*_1_ may vary spatially across the liver [60, 119]. Water ^1^H *R*_1_ is multiexponential, particularly with sequences where macromolecule-associated fast-relaxing water contributes to the measurement. Other substances in the liver may also contribute to the ^1^H signal, such as glycogen [87] or triglyceride [76, 131]. Inflowing blood [110, 132], physiologic motion [71], magnetization transfer, and iron affect the measured *R*_1_ in ways which depend both on the sequence and on the analysis employed. There may be systematic differences in *R*_1_ between fat-suppressed vs. non-fat-suppressed acquisitions; 2D acquisitions more vulnerable to inflow effects than 3D; breathhold or gated vs. free-breathing; and so on. Some investigators advocate the use of a “corrected” *T*_1_ to avoid bias caused by the relaxivity of iron-containing substances [65]. Because of these biases in the literature, studies which deviate from these survey data should not immediately be considered “incorrect”, but if large deviations are observed, then an explanation on methodological or physiological grounds should be sought.

There are some other limitations. While some publications reported carefully designed and conducted biomarker validation studies, in other publications, the precise value of *T*_1_ was only of incidental interest and possibly acquired with less care. However, in this survey, the study design and objectives were not incorporated into the weightings. Most studies did not report validation of their liver *R*_1_ by means of a phantom, so accuracy is unknown. It was difficult to explore the effect of methodology on *R*_1_, because some studies used methodology which was poorly described or did not appear robust, and because of correlation between field strength and methodology (old studies used old methodology and lower fields). Likewise, there was correlation between field strength and species (humans at low-medium fields, rats at medium–high fields and mice at high fields), so it was difficult to compare between species.

## Conclusion

Quantitative relaxometry requires validation with phantoms and analysis of propagation of errors. However, it is also good scientific practice to compare one’s own findings with prior literature. An investigator who finds their average liver *R*_1_ in normal liver to be within 8% of the fit to Eq. 3, with between-subject CoV < 8%, can conclude that their measurements are in agreement with the majority of the literature: for measurements far outside these limits, a physiological or methodological explanation should be sought.

## Supplementary Information

Below is the link to the electronic supplementary material.

Supplementary file1 (PDF 315 KB)

## Declarations

### Research involving human and animal participants

Not applicable, as this is a survey of previously published research.

## References

[1] Smith FW, Mallard JR, Reid A, Hutchison JMS (1981) Nuclear magnetic resonance tomographic imaging in liver disease. Lancet 317:963–966 10.1016/s0140-6736(81)91731-1 6112385

[2] Banerjee R, Pavlides M, Tunnicliffe EM, Piechnik SK, Sarania N, Philips R, Collier JD, Booth JC, Schneider JE, Wang LM, Delaney DW, Fleming KA, Robson MD, Barnes E, Neubauer S (2014). Multiparametric magnetic resonance for the non-invasive diagnosis of liver disease. J Hepatol.

[3] Haimerl M, Utpatel K, Verloh N, Zeman F, Fellner C, Nickel D, Teufel A, Fichtner-Feigl S, Evert M, Stroszczynski C, Wiggermann P (2017) Gd-EOB-DTPA-enhanced MR relaxometry for the detection and staging of liver fibrosis. Sci Rep 7:41429 10.1038/srep41429 PMC5269752 28128291

[4] Haimerl M, Verloh N, Fellner C, Zeman F, Teufel A, Fichtner-Feigl S, Schreyer AG, Stroszczynski C, Wiggermann P (2014) MRI-based estimation of liver function: Gd-EOB-DTPA-enhanced T1 relaxometry of 3T vs The MELD score. Sci Rep 4:5621 10.1038/srep05621 PMC4085628 25001391

[5] Luetkens JA, Klein S, Träber F, Schmeel FC, Sprinkart AM, Kuetting DLR, Block W, Uschner FE, Schierwagen R, Hittatiya K, Kristiansen G, Gieseke J, Schild HH, Trebicka J, Kukuk GM (2018) Quantification of liver fibrosis at T_1_ and T_2_ mapping with extracellular volume fraction MRI: Preclinical results. Radiology 288:748–754 10.1148/radiol.2018180051 29944086

[6] Li Z, Sun J, Chen L, Huang N, Hu P, Hu X, Han G, Zhou Y, Bai W, Niu T, Yang X (2016). Assessment of liver fibrosis using pharmacokinetic parameters of dynamic contrast-enhanced magnetic resonance imaging. J Magn Reson Imaging.

[7] O’Connor JPB, Naish JH, Jackson A, Waterton JC, Watson Y, Cheung S, Buckley DL, McGrath DM, Buonaccorsi GA, Mills SJ, Roberts C, Jayson GC, Parker GJM (2009) Comparison of normal tissue R_1_ and R_2_* modulation by oxygen and carbogen. Magn Reson Med 61:75–83 10.1002/mrm.21815 19097212

[8] Ziemian S, Green C, Sourbron S, Jost G, Schütz G, Hines CDG (2021) Ex vivo gadoxetate relaxivities in rat liver tissue and blood at five magnetic field strengths from 1.41 to 7T. NMR Biomed. 34:e4401 10.1002/nbm.4401 PMC7757196 32851735

[9] Raunig DL, McShane LM, Pennello G, Gatsonis C, Carson PL, Voyvodic JT, Wahl RL, Kurland BF, Schwarz AJ, Gönen M, Zahlmann G, Kondratovich MV, O’Donnell K, Petrick N, Cole PE, Garra B, Sullivan DC (2015). Quantitative imaging biomarkers: A review of statistical methods for technical performance assessment. Stat Methods Med Res.

[10] Hozo SP, Djulbegovic B, Hozo I (2005) Estimating the mean and variance from the median, range, and the size of a sample. BMC Med Res Methodol 5:13 10.1186/1471-2288-5-13 PMC1097734 15840177

[11] Araya YT, Martínez-Santiesteban F, Handler WB, Harris CT, Chronik BA, Scholl TJ (2017) Nuclear magnetic relaxation dispersion of murine tissue for development of T_1_ (R_1_) dispersion contrast imaging. NMR Biomed 30:e3789 10.1002/nbm.3789 29044888

[12] Diakova G, Korb JP, Bryant RG (2012) The magnetic field dependence of water T_1_ in tissues. Magn Reson Med 68:272–277 10.1002/mrm.23229 PMC3297724 22144333

[13] Thomsen C (1996). Quantitative magnetic resonance methods for in vivo investigation of the human liver and spleen. Technical aspects and preliminary clinical results. Acta Radiol Suppl.

[14] Keevil SF, Dolke G, Brooks AP, Armstrong P, Farthing MJG, Alstead EM, Smith MA (1992). Proton NMR relaxation times in the normal human liver at 0.08 T. Clin Radiol.

[15] Henriksen O, de Certaines JD, Spisni A, Cortsen M, Muller RN, Ring PB (1993). V. In vivo field dependence of proton relaxation times in human brain, liver and skeletal muscle: A multicenter study. Magn Reson Imaging.

[16] R Development Core Team (2018) R 3.5.1., A language and environment for statistical computing. R Found Stat Comput 2. https://www.R-project.org.

[17] Krynicki K (1966). Proton spin-lattice relaxation in pure water between 0°C and 100°C. Physica.

[18] Kamimura K, Fukukura Y, Yoneyama T, Takumi K, Tateyama A, Umanodan A, Shindo T, Kumagae Y, Ueno SI, Koriyama C, Nakajo M (2014). Quantitative evaluation of liver function with T1 relaxation time index on Gd-EOB-DTPA-Enhanced MRI: Comparison with signal intensity-based indices. J Magn Reson Imaging.

[19] Kim KA, Park MS, Kim IS, Kiefer B, Chung WS, Kim MJ, Kim KW (2012). Quantitative evaluation of liver cirrhosis using T1 relaxation time with 3 tesla MRI before and after oxygen inhalation. J Magn Reson Imaging.

[20] Heye T, Yang SR, Bock M, Brost S, Weigand K, Longerich T, Kauczor HU, Hosch W (2012). MR relaxometry of the liver: Significant elevation of T1 relaxation time in patients with liver cirrhosis. Eur Radiol.

[21] Block W, Reichel C, Träber F, Skodra T, Lamerichs R, Kreft B, Spengler U, Sauerbruch T, Schild H (1997) Effect of cytochrome P450 induction on phosphorus metabolites and proton relaxation times measured by in vivo ^31^P-magnetic resonance spectroscopy and ^1^H-magnetic resonance relaxometry in human liver. Hepatology 26:1587–1591 10.1002/hep.510260629 9398002

[22] de Certaines JD, Henriksen O, Spisni A, Cortsen M, Ring PB (1993). IV. In vivo measurements of proton relaxation times in human brain, liver, and skeletal muscle: A multicenter MRI study. Magn Reson Imaging.

[23] Van Lom KJ, Brown JJ, Perman WH, Sandstrom JC, Lee JKT (1991). Liver imaging at 1.5 Tesla: Pulse sequence optimization based on improved measurement of tissue relaxation times. Magn Reson Imaging.

[24] Steudel A, Harder T, Träber F, Dewes W, Schlolaut KH, Koster O (1989) Relaxationszeitmessungen in Der Kernspintomographischen Differentialdiagnose Von Lebertumoren. RöFo Fortschritte auf dem Gebiete der Röntgenstrahlen und der Neuen Bildgeb Verfahren 151:449–455 10.1055/s-2008-1047213 2554383

[25] Richards MA, Webb JAW, Jewell SE, Gregory WM, Reznek RH (1988). In-vivo measurement of spin lattice relaxation time (T1) of liver in healthy volunteers: The effects of age, sex and oral contraceptive usage. Br J Radiol.

[26] Thomsen C, Christoffersen P, Henriksen O, Juhl E (1990). Prolonged T1 in patients with liver cirrhosis: An in vivo MRI study. Magn Reson Imaging.

[27] Cassinotto C, Feldis M, Vergniol J, Mouries A, Cochet H, Lapuyade B, Hocquelet A, Juanola E, Foucher J, Laurent F, De Ledinghen V (2015). MR relaxometry in chronic liver diseases: Comparison of T1 mapping, T2 mapping, and diffusion-weighted imaging for assessing cirrhosis diagnosis and severity. Eur J Radiol.

[28] Henninger B, Kremser C, Rauch S, Eder R, Zoller H, Finkenstedt A, Michaely HJ, Schocke M (2012). Evaluation of MR imaging with T1 and T2* mapping for the determination of hepatic iron overload. Eur Radiol.

[29] Weinreb JC, Brateman L, Maravilla KR (1984). Magnetic resonance imaging of hepatic lymphoma. Am J Roentgenol.

[30] Belt TG, Cohen MD, Smith JA, Cory DA, McKenna S, Weetman R (1986). MRI of Wilms’ tumor: Promise as the primary imaging method. Am J Roentgenol.

[31] Ohtomo K, Itai Y, Furui S, Yoshikawa K, Yashiro N, Iio M (1985). Magnetic resonance imaging (MRI) of primary liver cancer. MRI- pathologic correlation. Radiat Med - Med Imaging Radiat Oncol.

[32] Nyman R, Ericsson A, Hemmingsson A, Jung B, Sperber G, Thuomas KÅ (1986). T1, T2, and relative proton density at 0.35 T for spleen, liver, adipose tissue, and vertebral body: Normal values. Magn Reson Med.

[33] Stark DD, Moseley ME, Bacon BR (1985). Magnetic resonance imaging and spectroscopy of hepatic iron overload. Radiology.

[34] Gilligan LA, Dillman JR, Tkach JA, Xanthakos SA, Gill JK, Trout AT (2019). Magnetic resonance imaging T1 relaxation times for the liver, pancreas and spleen in healthy children at 1.5 and 3 tesla. Pediatr Radiol.

[35] Kim JE, Kim HO, Bae K, Choi DS, Nickel D (2019). T1 mapping for liver function evaluation in gadoxetic acid–enhanced MR imaging: comparison of look-locker inversion recovery and B1 inhomogeneity–corrected variable flip angle method. Eur Radiol.

[36] Yang L, Ding Y, Rao S, Chen C, Zeng M (2020) T_1_ mapping on Gd-EOB-DTPA-enhanced MRI for the prediction of oxaliplatin-induced liver injury in a mouse model. J Magn Reson Imaging 53:896–902 10.1002/jmri.27377 32979019

[37] Bradley CR, Cox EF, Scott RA, James MW, Kaye P, Aithal GP, Francis ST, Guha IN (2018). Multi-organ assessment of compensated cirrhosis patients using quantitative magnetic resonance imaging. J Hepatol.

[38] Zhou ZP, Long LL, Qiu WJ, Cheng G, Huang LJ, Yang TF, Huang ZK (2017). Comparison of 10- and 20-min hepatobiliary phase images on Gd-EOB-DTPA-enhanced MRI T1 mapping for liver function assessment in clinic. Abdom Radiol.

[39] Agrawal S, Hoad CL, Francis ST, Guha IN, Kaye P, Aithal GP (2017). Visual morphometry and three non-invasive markers in the evaluation of liver fibrosis in chronic liver disease. Scand J Gastroenterol.

[40] Tunnicliffe EM, Banerjee R, Pavlides M, Neubauer S, Robson MD (2017). A model for hepatic fibrosis: the competing effects of cell loss and iron on shortened modified Look-Locker inversion recovery T1 (shMOLLI-T1) in the liver. J Magn Reson Imaging.

[41] Chen Y, Jiang Y, Pahwa S, Ma D, Lu L, Twieg MD, Wright KL, Seiberlich N, Griswold MA, Gulani V (2016). MR fingerprinting for rapid quantitative abdominal imaging. Radiology.

[42] Ding Y, Rao SX, Chen C, Li R, Zeng MS (2015). Assessing liver function in patients with HBV-related HCC: a comparison of T1 mapping on Gd-EOB-DTPA-enhanced MR imaging with DWI. Eur Radiol.

[43] Haimerl M, Verloh N, Zeman F, Fellner C, Müller-Wille R, Schreyer AG, Stroszczynski C, Wiggermann P (2013) Assessment of clinical signs of liver cirrhosis using T1 mapping on Gd-EOB-DTPA-enhanced 3T MRI. PLoS ONE 8:e85658 10.1371/journal.pone.0085658 PMC3877368 24392025

[44] Katsube T, Okada M, Kumano S, Hori M, Imaoka I, Ishii K, Kudo M, Kitagaki H, Murakami T (2011). Estimation of liver function using T1 mapping on Gd-EOB-DTPA-enhanced magnetic resonance imaging. Invest Radiol.

[45] Jafari F, Nayeri N, Tahsini M, Khodadoust AA (1999) Differentiation of hepatic cavernous hemangioma from metastases by rare sequence MR imaging. Magn Reson Imaging 17:669–677 10.1016/s0730-725x(99)00008-9 10372520

[46] Halavaara J, Lukkarinen S, Sepponen R, Markkola A, Tanttu J (2003). Contrast-to-noise ratio of multiple slice spin lock technique: Prospects for liver imaging. Br J Radiol.

[47] Skjold A, Vangberg TR, Kristoffersen A, Haraldseth O, Jynge P, Larsson HBW (2004). Relaxation enhancing properties of MnDPDP in human myocardium. J Magn Reson Imaging.

[48] De Bazelaire CMJ, Duhamel GD, Rofsky NM, Alsop DC (2004). MR Imaging Relaxation Times of Abdominal and Pelvic Tissues Measured in Vivo at 3.0 T: Preliminary Results. Radiology.

[49] Tadamura E, Hatabu H, Li W, Prasad PV, Edelman RR (1997). Effect of oxygen inhalation on relaxation times in various tissues. J Magn Reson Imaging.

[50] Morio S, Oh H, Endo N, Kawano E, Nakamura H, Asai T, Saito Y, Uchida Y, Ikehira H, Yoshida K (1997). Magnetic resonance imaging of reticulo-endothelial system in patients with idiopathic thrombocytopenic purpura. Am J Hematol.

[51] Tamburrini O, Andò S, Della Sala M, Maggiolini M, Sessa M (1993) Emocromatosi epatica secondaria: diagnosi e quantificazione con risonanza magnetica 0.5 T. Valore e limite Radiol Medica 86:841–846 8296005

[52] Blüml S, Schad LR, Stepanow B, Lorenz WJ (1993). Spin-lattice relaxation time measurement by means of a TurboFLASH technique. Magn Reson Med.

[53] Patrizio G, Pavone P, Testa A, Marsili L, Tettamanti E, Passariello R (1990). MR characterization of hepatic lesions by T-null inversion recovery sequence. J Comput Assist Tomogr.

[54] Squillaci E, Cecconi L, Tipaldi L, Grandinetti ML, Orlacchio A, Squillaci S (1989) La Risonanza Magnetica Nelle Lesioni Epatiche. Esperienza Con Campo Magnetico Da 1,5 T. Radiol Medica 78:585–592 2560578

[55] Rummeny E, Weissleder R, Stark DD, Saini S, Compton CC, Bennett W, Hahn PF, Wittenberg J, Malt RA, Ferrucci JT (1989). Primary liver tumors: Diagnosis by MR imaging. Am J Roentgenol.

[56] Rademaker M, Webb JAW, Lowe DG, Meyrick-thomas RH, Kirby JDT, Munro DD (1987) Magnetic resonance imaging as a screening procedure for methotrexate induced liver damage. Br J Dermatol 117:311–316 10.1111/j.1365-2133.1987.tb04137.x 3676081

[57] The Clinical NMR Group (1987). Magnetic resonance imaging of parenchymal liver disease: a comparison with ultrasound, radionuclide scintigraphy and X-ray computed tomography. Clin Radiol.

[58] Richards MA, Webb J, Reznek RH, Davies G, Jewell SE, Shand WS, Wrigley PFM, Lister TA (1986). Detection of spread of malignant lymphoma to the liver by low field strength magnetic resonance imaging. Br Med J (Clin Res Ed).

[59] Glazer GM, Aisen AM, Francis IR, Gyves JW, Lande I, Adler DD (1985). Hepatic cavernous hemangioma: Magnetic resonance imaging. Radiology.

[60] Obmann VC, Mertineit N, Marx C, Berzigotti A, Ebner L, Heverhagen JT, Christe A, Huber AT (2019). Liver MR relaxometry at 3T – segmental normal T1 and T2* values in patients without focal or diffuse liver disease and in patients with increased liver fat and elevated liver stiffness. Sci Rep.

[61] Doyle FH, Pennock JM, Banks LM, McDonnell MJ, Bydder GM, Steiner RE, Young IR, Clarke GJ, Pasmore T, Gilderdale DJ (1982). Nuclear magnetic resonance imaging of the liver: Initial experience. Am J Roentgenol.

[62] Ramachandran P, Serai SD, Veldtman GR, Lang SM, Mazur W, Trout AT, Dillman JR, Fleck RJ, Taylor MD, Alsaied T, Moore RA (2019). Assessment of liver T1 mapping in fontan patients and its correlation with magnetic resonance elastography-derived liver stiffness. Abdom Radiol.

[63] Huber AT, Razakamanantsoa L, Lamy J, Giron A, Cluzel P, Kachenoura N, Redheuil A (2020). Multiparametric differentiation of idiopathic dilated cardiomyopathy with and without congestive heart failure by means of cardiac and hepatic T1-weighted MRI mapping. Am J Roentgenol.

[64] Obmann VC, Marx C, Berzigotti A, Mertineit N, Hrycyk J, Gräni C, Ebner L, Ith M, Heverhagen JT, Christe A, Huber AT (2019). Liver MRI susceptibility-weighted imaging (SWI) compared to T2* mapping in the presence of steatosis and fibrosis. Eur J Radiol.

[65] Mojtahed A, Kelly CJ, Herlihy AH, Kin S, Wilman HR, McKay A, Kelly M, Milanesi M, Neubauer S, Thomas EL, Bell JD, Banerjee R, Harisinghani M (2019). Reference range of liver corrected T1 values in a population at low risk for fatty liver disease—a UK Biobank sub-study, with an appendix of interesting cases. Abdom Radiol.

[66] Chen Y, Lee GR, Aandal G, Badve C, WrighT KL, Griswold MA, Seiberlich N, Gulani V (2016) Rapid volumetric T_1_ mapping of the abdomen using three-dimensional through-time spiral GRAPPA. Magn Reson Med 75:1457–1465 10.1002/mrm.25693 PMC4651863 25980949

[67] Wiese S, Voiosu A, Hove JD, Danielsen KV, Voiosu T, Grønbæk H, Møller HJ, Genovese F, Reese-Petersen AL, Mookerjee RP, Clemmesen JO, Gøtze JP, Andersen O, Møller S, Bendtsen F (2020). Fibrogenesis and inflammation contribute to the pathogenesis of cirrhotic cardiomyopathy. Aliment Pharmacol Ther.

[68] Runge VM, Clanton JA, Smith FW, Hutchison J, Mallard J, Partain CL, James AE (1983). Nuclear magnetic resonance of iron and copper disease states. AJR Am J Roentgenol.

[69] Ebara M, Ohto M, Watanabe Y, Kimura K, Saisho H, Tsuchiya Y, Okuda K, Arimizu N, Kondo F, Ikehira H (1986). Diagnosis of small hepatocellular carcinoma: Correlation of MR imaging and tumor histologic studies. Radiology.

[70] Brasch RC, Wesbey GE, Gooding CA, Koerper MA (1984). Magnetic resonance imaging of transfusional hemosiderosis complicating thalassemia major. Radiology.

[71] Ehman RL, McNamara MT, Pallack M, Hricak H, Higgins CB (1984) Magnetic resonance imaging with respiratory gating: Techniques and advantages. Am J Roentgenol 143:1175–1182 10.2214/ajr.143.6.1175 6333787

[72] Rödl W (1985) Differentialdiagnose von Lebererkrankungen im Kernspintomogramm. RöFo Fortschritte auf dem Gebiete der Rontgenstrahlen und der bildgeb Verfahren 142:505–510 10.1055/s-2008-1052696 2988031

[73] Rupp N, Reiser M, Stetter E (1983). The diagnostic value of morphology and relaxation times in NMR-imaging of the body. Eur J Radiol.

[74] Buonocore E, Borkowski GP, Pavlicek W, Ngo F (1983). NMR imaging of the abdomen: Technical considerations. Am J Roentgenol.

[75] Brown DW, Henkelman RM, Poon PY, Fisher MM (1985). Nuclear magnetic resonance study of iron overload in liver tissue. Magn Reson Imaging.

[76] Mozes FE, Tunnicliffe EM, Moolla A, Marjot T, Levick CK, Pavlides M, Robson MD (2019) Mapping tissue water T_1_ in the liver using the MOLLI T_1_ method in the presence of fat, iron and B_0_ inhomogeneity. NMR Biomed 32:e4030 10.1002/nbm.4030 PMC6492199 30462873

[77] Ding Y, Rao SX, Zhu T, Chen CZ, Li RC, Zeng MS (2015). Liver fibrosis staging using T1 mapping on gadoxetic acid-enhanced MRI compared with DW imaging. Clin Radiol.

[78] Moss AA, Goldberg HI, Stark DB, Davis PL, Margulis AR, Kaufman L, LEC, (1984). Hepatic tumors: Magnetic resonance and CT appearance. Radiology.

[79] Träber F, Steudel A, Harder T (1990) In-vivo-messung von geweberelaxationszeiten mit lokalisierter ^31^P- Und ^1^H-MR-Spektroskopie. RöFo Fortschritte auf dem Gebiete der Rontgenstrahlen und der Neuen Bildgeb Verfahren 153:209–215 10.1055/s-2008-1033363 2168079

[80] Wang C, Wang ZC, Ding Y, Zeng MS, Rao SX (2018). Value of gadoxetate disodium-enhanced magnetic resonance on hepatobiliary phase T1 mapping for predicting liver injury. Zhonghua Gan Zang Bing Za Zhi.

[81] Ehman RL, Kjos BO, Hricak H, Brasch RC, Higgins CB (1985). Relative intensity of abdominal organs in MR images. J Comput Assist Tomogr.

[82] Flak B, Ajzen S, Li DKB, Cooperberg PL, Clark C (1989). Hemangioma of the liver: Characteristics exhibited on a 0.15 Tesla scanner. Can Assoc Radiol J.

[83] Fletcher BD, Kopiwoda SY, Strandjord SE, Nelson AD, Pickering SP (1985). Abdominal neuroblastoma: Magnetic resonance imaging and tissue characterization. Radiology.

[84] Foley WD, Kneeland JB, Cates JD, Kellman GM, Lawson TL, Middleton WD, Hendrick RE (1987). Contrast optimization for the detection of focal hepatic lesions by MR imaging at 1.5 T. Am J Roentgenol.

[85] Schmidt HC, Tscholakoff D, Hricak H, Higgins CB (1985). Mr image contrast and relaxation times of solid tumors in the chest, abdomen, and pelvis. J Comput Assist Tomogr.

[86] Rödl W (1984) Differential diagnosis of liver diseases with the aid of nuclear magnetic resonance imaging. In: Demling L, Lutz H, Wenz W, Wildhirt E (eds) Diagnostic Imaging Methods in Hepatolology: proceedings of the 37th Falk Symposium, held during Basel Liver Week, Basel, September 29–October 2, 1983. MTP Press, Lancaster, MA USA, pp 153–158

[87] Weis J, Kullberg J, Ahlström H (2018). Multiple breath-hold proton spectroscopy of human liver at 3T: Relaxation times and concentrations of glycogen, choline, and lipids. J Magn Reson Imaging.

[88] Hoad CL, Palaniyappan N, Kaye P, Chernova Y, James MW, Costigan C, Austin A, Marciani L, Gowland PA, Guha IN, Francis ST, Aithal GP (2015). A study of T1 relaxation time as a measure of liver fibrosis and the influence of confounding histological factors. NMR Biomed.

[89] O’Connor JPB, Jackson A, Buonaccorsi GA, Buckley DL, Roberts C, Watson Y, Cheung S, McGrath DM, Naish JH, Rose CJ, Dark PM, Jayson GC, Parker GJM (2007). Organ-specific effects of oxygen and carbogen gas inhalation on tissue longitudinal relaxation times. Magn Reson Med.

[90] Nyman R, Rhen S, Ericsson A, Glimelius B, Hagberg H, Hemmingsson A, Sundström C (1987) An attempt to characterize malignant lymphoma in spleen, liver and lymph nodes with magnetic resonance imaging. Acta Radiol 28:527–533 2960343

[91] Hardy CJ, Edelstein WA, Vatis D, Harms R, Adams WJ (1985). Calculated T1 images derived from a partial saturation-inversion recovery pulse sequence with adiabatic fast passage. Magn Reson Imaging.

[92] Kinami Y, Yokota H, Takata M, Takashima S, Yamamoto I (1988). Magnetic resonance imaging in the diagnosis of tumors of the liver. Gastroenterol Jpn.

[93] Leung A, Bydder G, Steiner R, Bryant D, Young I (1984). Magnetic resonance imaging of the kidneys. AJR Am J Roentgenol.

[94] Okada M, Murakami T, Yada N, Numata K, Onoda M, Hyodo T, Inoue T, Ishii K, Kudo M (2015). Comparison between T1 relaxation time of Gd-EOB-DTPA-enhanced MRI and liver stiffness measurement of ultrasound elastography in the evaluation of cirrhotic liver. J Magn Reson Imaging.

[95] Chow AM, Gao DS, Fan SJ, Qiao Z, Lee FY, Yang J, Man K, Wu EX (2012). Measurement of liver T1 and T2 relaxation times in an experimental mouse model of liver fibrosis. J Magn Reson Imaging.

[96] Ding Y, Yang L, Rao SX, Zeng MS (2019). Gadoxetic disodium-enhanced MRI to characterize T1 relaxation values and expression level of organic anion transporters and multidrug resistance protein on hepatocyte surface membrane of normal C57BL/6 mice. Zhonghua Gan Zang Bing Za Zhi.

[97] Matsuo-Tezuka Y, Sasaki Y, Iwai T, Kurasawa M, Yorozu K, Tashiro Y, Hirata M (2019) T_2_* relaxation time obtained from magnetic resonance imaging of the liver is a useful parameter for use in the construction of a murine model of iron overload. Contrast Media Mol Imaging 2019:7463047 10.1155/2019/7463047 PMC6778918 31598113

[98] Faller TL, Trotier AJ, Miraux S, Ribot EJ (2019). Radial MP2RAGE sequence for rapid 3D T1 mapping of mouse abdomen: application to hepatic metastases. Eur Radiol.

[99] Anderson CE, Wang CY, Gu Y, Darrah R, Griswold MA, Yu X, Flask CA (2018). Regularly incremented phase encoding – MR fingerprinting (RIPE-MRF) for enhanced motion artifact suppression in preclinical cartesian MR fingerprinting. Magn Reson Med.

[100] Jackson LH, Vlachodimitropoulou E, Shangaris P, Roberts TA, Ryan TM, Campbell-Washburn AE, David AL, Porter JB, Lythgoe MF, Stuckey DJ (2017) Non-invasive MRI biomarkers for the early assessment of iron overload in a humanized mouse model of β-thalassemia. Sci Rep 7:43439 10.1038/srep43439 PMC5327494 28240317

[101] Eberhardt C, Wurnig MC, Wirsching A, Rossi C, Feldmane I, Lesurtel M, Boss A (2018) Prediction of small for size syndrome after extended hepatectomy: Tissue characterization by relaxometry, diffusion weighted magnetic resonance imaging and magnetization transfer. PLoS ONE 13:e0192847 10.1371/journal.pone.0192847 PMC5812661 29444146

[102] Li H, Gray BD, Corbin I, Lebherz C, Choi H, Lund-Katz S, Wilson JM, Glickson JD, Zhou R (2004). MR and fluorescent imaging of low-density lipoprotein receptors. Acad Radiol.

[103] Oostendorp M, Douma K, Hackeng TM, Post MJ, Van Zandvoort MAMJ, Backes WH (2010). Gadolinium-labeled quantum dots for molecular magnetic resonance imaging: R1 versus R2 mapping. Magn Reson Med.

[104] Ramasawmy R, Campbell-Washburn AE, Wells JA, Johnson SP, Pedley RB, Walker-Samuel S, Lythgoe MF (2015). Hepatic arterial spin labelling MRI: An initial evaluation in mice. NMR Biomed.

[105] Polasek M, Fuchs BC, Uppal R, Schühle DT, Alford JK, Loving GS, Yamada S, Wei L, Lauwers GY, Guimaraes AR, Tanabe KK, Caravan P (2012). Molecular MR imaging of liver fibrosis: A feasibility study using rat and mouse models. J Hepatol.

[106] Müller A, Hochrath K, Stroeder J, Hittatiya K, Schneider G, Lammert F, Buecker A, Fries P (2017) Effects of liver fibrosis progression on tissue relaxation times in different mouse models assessed by ultrahigh field magnetic resonance imaging. Biomed Res Int 2017:8720367 10.1155/2017/8720367 PMC5286538 28194423

[107] Braren R, Curcic J, Remmele S, Altomonte J, Ebert O, Rummeny EJ, Steingoetter A (2011) Free-breathing quantitative dynamic contrast-enhanced magnetic resonance imaging in a rat liver tumor model using dynamic radial T_1_ mapping. Invest Radiol 46:624–631 10.1097/RLI.0b013e31821e30e7 21577121

[108] Cheng HLM, Haedicke IE, Cheng W, Nofiele JT, Zhang XA (2014). Gadolinium-free T1 contrast agents for MRI: Tunable pharmacokinetics of a new class of manganese porphyrins. J Magn Reson Imaging.

[109] Nekolla S, Gneiting T, Syha J, Deichmann R, Haase A (1992) T1 maps by k-space reduced snapshot-FLASH MRI. J Comput Assist Tomogr 16:327–332 10.1097/00004728-199203000-00031 1545039

[110] Chouhan MD, Ramasawmy R, Bainbridge A, Campbell-Washburn A, Halligan S, Davies N, Walker-Samuel S, Lythgoe MF, Mookerjee RP, Taylor SA (2020) Liver perfusion MRI in a rodent model of cirrhosis: Agreement with bulk-flow phase-contrast MRI and noninvasive evaluation of inflammation in chronic liver disease using flow-sensitive alternating inversion recovery arterial spin labelling and tissue T_1_. NMR Biomed 34:e4423 10.1002/nbm.4423 PMC8427466 33029872

[111] Marzola P, Maggioni F, Vicinanza E, Daprà M, Cavagna FM (1997). Evaluation of the hepatocyte-specific contrast agent gadobenate dimeglumine for MR imaging of acute hepatitis in a rat model. J Magn Reson Imaging.

[112] Hazle JD, Narayana PA, Dunsford HA (1991) In vivo NMR, biochemical, and histologic evaluation of alcohol-induced fatty liver in rat and a comparison with CCl4 hepatotoxicity. Magn Reson Med 19:124–135 10.1002/mrm.1910190112 2046527

[113] Hazle JD, Narayana PA, Dunsford HA (1990). Chronic carbon tetrachloride and phospholipase D hepatotoxicity in rat: In vivo 1H magnetic resonance, total lipid analysis, and histology. Magn Reson Med.

[114] Ling M, Brauer M (1992). Ethanol-induced fatty liver in the rat examined by in vivo 1H chemical shift selective magnetic resonance imaging and localized spectroscopic methods. Magn Reson Imaging.

[115] Herfkens R, Davis P, Crooks L, Kaufman L, Price D, Miller T, Margulis AR, Watts J, Hoenninger J, Arakawa M, McRee R (1981). Nuclear magnetic resonance imaging of the abnormal live rat and correlations with tissue characteristics. Radiology.

[116] Davis PL, Kaufman L, Crooks LE, Miller TR (1981). Detectability of hepatomas in rat livers by nuclear magnetic resonance imaging. Invest Radiol.

[117] Hoy AM, McDonald N, Lennen RJ, Milanesi M, Herlihy AH, Kendall TJ, Mungall W, Gyngell M, Banerjee R, Janiczek RL, Murphy PS, Jansen MA, Fallowfield JA (2018). Non-invasive assessment of liver disease in rats using multiparametric magnetic resonance imaging: a feasibility study. Biol Open.

[118] Zhou IY, Jordan VC, Rotile NJ, Akam E, Krishnan S, Arora G, Krishnan H, Slattery H, Warner N, Mercaldo N, Farrar CT, Wellen J, Martinez R, Schlerman F, Tanabe KK, Fuchs BC, Caravan P (2020). Advanced MRI of liver fibrosis and treatment response in a rat model of nonalcoholic steatohepatitis. Radiology.

[119] Li J, Liu H, Zhang C, Yang S, Wang Y, Chen W, Li X, Wang D (2020). Native T1 mapping compared to ultrasound elastography for staging and monitoring liver fibrosis: an animal study of repeatability, reproducibility, and accuracy. Eur Radiol.

[120] Gao Y, Erokwu BO, Desantis DA, Croniger CM, Schur RM, Lu L, Mariappuram J, Dell KM, Flask CA (2016). Initial evaluation of hepatic T1 relaxation time as an imaging marker of liver disease associated with autosomal recessive polycystic kidney disease (ARPKD). NMR Biomed.

[121] Gambarota G, Veltien A, Van Laarhoven H, Philippens M, Jonker A, Mook OR, Frederiks WM, Heerschap A (2004). Measurements of T1 and T2 relaxation times of colon cancer metastases in rat liver at 7 T. Magn Reson Mater Physics, Biol Med.

[122] Fan YD, Vanzieleghem B, Achten E, De Deene Y, Defreyne L, Praet M, Van Huysse J, Kunnen M, De Hemptinne B (2001). T1 relaxation times for viability evaluation of the engrafted and the native liver in a rat model of heterotopic auxiliary liver transplantation: A pilot study. NMR Biomed.

[123] Nakakoshi T, Kajiyama M, Fujita N, Jong-Hon K, Takeichi N, Miyasaka K (1996). Quantitative analyses of correlations of signal intensity on T1-weighted images and T1 relaxation time with copper concentration in the rat liver. Acad Radiol.

[124] Chamuleau RAFM, De Nie JHNCI, Moerland MA, Van der Lende OR, Smidt J (1988). Is the magnetic resonance imaging proton spin-lattice relaxation time a reliable noninvasive parameter of developing liver fibrosis?. Hepatology.

[125] Ganesh T, Estrada M, Yeger H, Duffin J, Margaret Cheng HL (2017) A non-invasive magnetic resonance imaging approach for assessment of real-time microcirculation dynamics. Sci Rep 7:7468 10.1038/s41598-017-06983-6 PMC5547069 28784990

[126] Sheng RF, Wang HQ, Yang L, Jin KP, Xie YH, Fu CX, Zeng MS (2017). Assessment of liver fibrosis using T1 mapping on Gd-EOB-DTPA-enhanced magnetic resonance. Dig Liver Dis.

[127] Soares AF, Lei H (2018) Non-invasive diagnosis and metabolic consequences of congenital portosystemic shunts in C57BL/6 J mice. NMR Biomed 31:e3873 10.1002/nbm.3873 29266459

[128] Steudel A, Traber F, Krahe T, Schiffmann O, Harder T (1990) Qualitatskontrolle der quantitativen mr-tomographie: in-vitro und in-vivo-uberprufung von relaxationszeitmessungen. RoFo Fortschritte auf dem Gebiete der Rontgenstrahlen und der Neuen Bildgeb Verfahren 152:673–676 10.1055/s-2008-1046946 2163072

[129] Bachtiar V, Kelly MD, Wilman HR, Jacobs J, Newbould R, Kelly CJ, Gyngell ML, Groves KE, McKay A, Herlihy AH, Fernandes CC, Halberstadt M, Maguire M, Jayaratne N, Linden S, Neubauer S, Banerjee R (2019) Repeatability and reproducibility of multiparametric magnetic resonance imaging of the liver. PLoS ONE 14:e0214921 10.1371/journal.pone.0214921 PMC6457552 30970039

[130] Moher D, Liberati A, Tetzlaff J, Altman DG, Altman D, Antes G, Atkins D, Barbour V, Barrowman N, Berlin JA, Clark J, Clarke M, Cook D, D’Amico R, Deeks JJ, Devereaux PJ, Dickersin K, Egger M, Ernst E, Gøtzsche PC, Grimshaw J, Guyatt G, Higgins J, Ioannidis JPA, Kleijnen J, Lang T, Magrini N, McNamee D, Moja L, Mulrow C, Napoli M, Oxman A, Pham B, Rennie D, Sampson M, Schulz KF, Shekelle PG, Tovey D, Tugwell P (2009) Preferred reporting items for systematic reviews and meta-analyses: The PRISMA statement. PLoS Med 6:e1000097 10.1371/journal.pmed.1000097 PMC2707599 19621072

[131] Haimerl M, Probst U, Poelsterl S, Fellner C, Nickel D, Weigand K, Brunner SM, Zeman F, Stroszczynski C, Wiggermann P (2018) Evaluation of two-point Dixon water-fat separation for liver specific contrast-enhanced assessment of liver maximum capacity. Sci Rep 8:13863 10.1038/s41598-018-32207-6 PMC6138716 30218001

[132] Axel L (1984). Blood flow effects in magnetic resonance imaging. Am J Roentgenol.

