# Supplementary material for: Survey of water proton longitudinal relaxation in liver in vivo
Source: MAGMA. 2021 May 12;34(6):779–89. doi: 10.1007/s10334-021-00928-x (PMC8578172; doi:10.1007/s10334-021-00928-x)
Supplement: Supplementary file 1 — Supplementary file1 (PDF 315 KB) [file 10334_2021_928_MOESM1_ESM.pdf]

Survey of water proton longitudinal relaxation in liver in vivo

John C. Waterton.

Supplementary material

Supplementary material 1: search strategy ... 2
Supplementary material 2: justification for Eq.1 ... 5
Supplementary material 3: Extract from R console ... 6
Supplementary material 4: additional exploratory fitting ... 9
Supplementary material 5: common methods ... 11
Supplementary material 6: subgroup analyses ... 12
Analyses for specific species and fields ... 12
Does R_1 deviate for subgroups? ... 12
Supplementary material 7: early vs post-1992 studies ... 13
Supplementary material 8: variance components ... 14

Supplementary material 1: search strategy

The search was not PRISMA-compliant. The motivation for this work (as explained in the introduction) was imaging biomarker development in the liver. At the time of writing, liver R_1 is most commonly measured in man at 1.5T or 3T (with 7T possible in some centres), and in rodents at 4.7T, 7T, or 9.4T (with 3T, 11.7T, 14.1T and 21.1T possible in some centres), so these are the field strengths of interest.

There were known to be many published liver R_1 studies at 1.5T or 3T.

The aims of the search were threefold

- To gather a large, but not necessarily exhaustive, sample of the published 1.5T and 3T liver R_1 studies.
- To make a thorough survey of published liver R_1 studies at >3T.
- To include convenient and sufficient, but not necessarily exhaustive, coverage of the older liver R_1 literature at <1.5T to help constrain the B_0-dependence models

The main challenges found in pilot searching were

- It was difficult to find search terms which retrieved publications measuring T_1 without also retrieving an unmanageably large number of publications using T_1-weighted MRI
- Many quantitative studies with DCE-MRI, gadoxetate or other contrast agents did measure T_1 or R_1, but suitable keywords were absent from the title and abstract.
- Likewise, studies of relaxivity needed to measure R_1, but suitable keywords were absent from the title and abstract

The dataset evolved as follows:

July 2019.

- A pilot, performed as part of a separate project, and searching on keywords in titles only, yielded 107 abstracts from which 19 papers were included

March 2020.

- A broader search (see below) yielded 297 abstracts from which 143 papers of interest were extracted and reviewed

March 2020-December 2020.

- Focussed searches for liver MRI with “rats”, “mice”, “4.7T”, “7T”, “9.4T”, “11.7T”, “14.1T” or “21.1T” were conducted
- Further citations were extracted from the papers reviewed

January-March 2021

- A final search for recent publications (2019-2021) was conducted
- By this point approximately 500 abstracts had been read, from which approximately 270 papers were selected and reviewed, of which approximately 154 failed to meet the inclusion criteria, leaving 116 papers for analysis.

March 2020 search

```
--------------------------------------------------------------------------------
1     magnetic resonance imaging.mp. or Magnetic Resonance Imaging/ (466863)
2     Magnetic Resonance Imaging/ or mri.mp. (454367)
3     liver.mp. or Liver/ (1015372)
4     1 or 2 (502195)
5     3 and 4 (21312)
6     relaxation.mp. [mp=title, abstract, original title, name of substance word, subject
heading word, floating
sub-heading word, keyword heading word, organism supplementary concept word, protocol
supplementary concept word, rare
disease supplementary concept word, unique identifier, synonyms] (97261)
7     T1.mp. [mp=title, abstract, original title, name of substance word, subject heading
word, floating sub-heading
word, keyword heading word, organism supplementary concept word, protocol supplementary
concept word, rare disease
supplementary concept word, unique identifier, synonyms] (88412)
8     longitudinal.mp. [mp=title, abstract, original title, name of substance word, subject
heading word, floating
sub-heading word, keyword heading word, organism supplementary concept word, protocol
supplementary concept word, rare
disease supplementary concept word, unique identifier, synonyms] (255496)
9     spin-lattice.mp. [mp=title, abstract, original title, name of substance word, subject
heading word, floating
sub-heading word, keyword heading word, organism supplementary concept word, protocol
supplementary concept word, rare
disease supplementary concept word, unique identifier, synonyms] (2283)
10     spin lattice.mp. [mp=title, abstract, original title, name of substance word, subject
heading word, floating
sub-heading word, keyword heading word, organism supplementary concept word, protocol
supplementary concept word, rare
disease supplementary concept word, unique identifier, synonyms] (2283)
11     6 or 7 or 8 or 9 (429161)
12     5 and 11 (3092)
13     5 and 6 (664)
14     7 or 8 or 9 (341049)
15     13 and 14 (333)
16     limit 15 to (mice or rats) (125)
17     limit 15 to humans (196)
18     16 or 17 (297)
19     from 18 keep 3-4,8-9,11,13-14,16-20,25-29,31,33,35,37-41,43-45,47-48,50-51,54-59,62-
66,69-70,72-73,77-79,81-83,85,87,89-90,93,98-102,108,111,117,119,125,129-132,137,141-
142,145,152-154,160,162,169,175,177-179,181-184,186,188,190-191,195,197-
198,200,202,206,208,211-212,215,218,220,224-225,232-233,236-237,239-241,246,250,254-256,258-
261,265-267,269,271-273,279-280,283-286,289,293-297
(143)
20     16 and 19 (49)
21     17 and 19 (100)
22     21 not 20 (94)
```

Supplementary material 2: justification for Eq.1

Where T_1±SD was reported, a point estimate of R_1 was estimated as T_1^{-1} and the between-subject variance in R_1 was estimated as:

$$0.25\left(\left((T_1 - SD)^{-1}\right) - \left((T_1 + SD)^{-1}\right)\right)^2 \qquad \text{Eq.1}$$

The justification for this equation is that it is mathematically impossible for both R_1 and T_1 to be perfectly normally distributed, although, unless N and CoV are large, it is also impossible to detect a deviation from normality in reciprocal data from a normal distribution. For example, if T_1 is perfectly normally distributed with T_1=1.000±0.100 (mean±SD), then correspondingly R_1~1.010±0.104. The approximation Eq.1 yields R_1~1.000±0.101, corresponding to ~1% error in R_1, while the approximated R_1 CoV of 10.1% slightly underestimates the true R_1 CoV of 10.3%. However, the normal distribution is itself merely a mathematically-convenient approximation to the true biological distribution of the T_1 or R_1 data, which is unknown and perhaps unknowable. These tiny errors are generally at least an order of magnitude below the precision of the measurements, and so it was thought safe to neglect them.

Supplementary material 3: Extract from R console

```
> as.data.frame(R1data)
        omega        R1      weight      N
1  2514857486 1.0764263   52.6991614   7.00
2  2514857486 0.9832842   53.3181534  11.00
3  2514857486 0.7961783  764.9407085  10.00
4  2514857486 0.7352941  946.5877778  10.00
5  1872766213 1.1806375  759.9204043   6.00
6  1872766213 0.7570000  685.5493865   5.00
7  1872766213 1.0050251  289.3876126  12.00
8  1872766213 0.9803922  298.5984000  16.00
9  1872766213 0.8826125 1422.9160000   3.00
10 1872766213 1.0050251 1087.0758510   5.00
11 1872766213 1.3400000    0.0000000   1.00
12 1872766213 1.0162602   47.3116688   4.00
13 1872766213 1.0427529   63.2562088   4.00
14 1257428743 1.0298661  455.3946320   6.00
15 1257428743 1.3157895    0.0000000   1.00
16 1257428743 0.9523810    0.0000000   1.00
17 1257428743 1.2738854   27.0013724   4.00
18 1257428743 1.0106114  143.0876665  22.00
19  802614091 1.2121212   55.8382563  27.00
20  802614091 1.4925373   40.2318367  24.00
21  802614091 1.0626993   40.6392188  92.00
22  802614091 1.5171514  324.9174625  10.00
23  802614091 1.3947001  113.6822423   7.00
24  802614091 1.3404826    0.0000000  78.00
25  802614091 1.2820513   46.0982323  78.00
26  802614091 1.3089005   52.5207553  12.00
27  802614091 1.2515645  107.4776510  12.00
28  802614091 1.3020833  115.8067815  14.00
29  802614091 1.0651690   30.3862388  11.00
30  802614091 1.2565684  143.7991179  11.00
31  802614091 1.2947269  196.9513038  11.00
32  802614091 1.7301038  132.8634040  10.00
33  802614091 1.6863406  101.8233885  10.00
34  802614091 1.4265335 1235.8669940   2.00
35  802614091 1.8281536  455.4920990   6.00
36  802614091 1.3300000   15.7558574   3.00
37  802614091 1.2315271    0.0000000  38.00
38  802614091 1.7241379   82.5473197  18.00
39  802614091 1.3037810   64.8179095  11.00
40  802614091 1.7158545   86.0241528  38.00
41  802614091 1.3670540    0.0000000  10.00
42  802614091 1.3422819    9.8758578   8.00
43  802614091 1.3145787   52.5176092 176.00
44  802614091 1.3037810    0.0000000  96.00
45  802614091 1.1956002   71.8060686  16.00
46  802614091 1.2360939   14.7244862   6.00
47  802614091 1.2135922   19.6371273  26.00
48  802614091 1.3831259  102.9033157   5.00
49  802614091 1.0101010   83.6683820   5.00
50  802614091 1.3333333  122.5394558   7.00
51  802614091 1.3513514   63.6322835   5.00
52  802614091 1.1016856  119.3197839  51.00
53  802614091 1.1357183  178.2453864  51.00
54  802614091 1.1299435   82.2044444   4.00
55  535076061 1.4705882    8.6005338   3.00
56  535076061 1.1025358   50.5861325  10.00
57  535076061 1.1013216   36.9027759  10.00
58  401307046 2.3110700   12.5425934   7.00
59  401307046 1.6835017   33.8793844   9.00
60  401307046 1.7574692   68.2699655  12.00
61  401307046 1.5723270  162.8090220  40.00
62  401307046 1.5822785  115.7397977  24.00
63  401307046 1.4658458   25.2906120  53.00
64  401307046 1.3819790   25.0576635  68.00
65  401307046 1.6352345  142.0079848  10.00
66  401307046 1.7543860   28.6760250   5.00
```

```
67   401307046 1.6835017  100.9226901    7.00
68   401307046 1.5400000   57.4910253   15.00
69   401307046 1.7543860   56.4423735   11.00
70   401307046 1.6129032  119.8555760    7.00
71   401307046 1.7793594  248.0238975    5.00
72   401307046 1.5015015  133.9057659 1037.00
73   401307046 1.8382353   27.3379592    9.00
74   401307046 1.3326226   50.3682698   41.00
75   401307046 2.2222222  102.1110250   24.00
76   401307046 1.3774105   97.8483045   14.00
77   401307046 1.5733166    0.0000000   26.00
78   401307046 2.2123894    0.0000000    1.00
79   401307046 1.6891892    0.0000000    1.00
80   401307046 1.5600000    0.0000000    3.00
81   401307046 1.5552100  156.1439574   35.00
82   401307046 1.4749263  103.4329680   31.00
83   401307046 1.7145306   56.8432712    9.00
84   401307046 1.3034241  120.6442913    4.00
85   401307046 1.2939289  165.2959895    4.00
86   401307046 1.3888889   13.3001831   20.75
87   401307046 1.7605634   29.2594813   14.00
88   401307046 2.0366599   10.4295375   64.00
89   401307046 1.4652015    4.3031560    4.00
90   401307046 1.4814815    7.9802927    7.00
91   401307046 2.0000000    9.5206618   40.00
92   401307046 2.0080321    0.0000000    1.00
93   401307046 1.7064846   76.8432055    6.00
94   401307046 1.2500000    0.9452160   31.00
95   267538030 2.1008403   12.0843141   13.00
96   267538030 2.6525199   32.0374112   16.00
97   160522818 2.0491803    3.3489000   21.00
98   160522818 2.3310023    4.0135114    6.00
99   160522818 2.2222222    2.8191599   16.00
100  160522818 2.0040080    5.4717924   15.00
101  133769015 2.4857072   65.1614542   10.00
102  133769015 2.4330900    2.3623988   40.00
103  133769015 2.6595745  199.5890818    7.00
104  133769015 2.7932961   50.4415605   20.75
105  133769015 4.0650407  146.3664432   15.00
106  133769015 1.5360983   12.2866428    7.00
107  133769015 2.1097046    5.3957295   17.00
108   93638311 1.9801980    1.7179436   20.00
109   93638311 2.2573363    6.2811572   14.00
110   93638311 2.5252525    2.8348440    5.00
111   93638311 2.7322404    0.7069127   25.00
112   93638311 3.7453184    1.8139336    5.00
113   93638311 1.9801980    0.0000000    7.00
114   93638311 1.8761726    3.8137782   28.00
115   93638311 2.2172949    1.5554019   15.00
116   93638311 2.3923445    0.4836493   10.00
117   93638311 2.3255814   20.9993063   30.00
118   93638311 2.5188917    1.4501779   14.00
119   93638311 2.6525199    3.2188609   35.00
120   93638311 2.2624434    8.1161207   14.00
121   93638311 2.8490028    2.8561483   61.00
122   80261409 3.1525851    4.3451049    8.00
123   69559888 4.7393365    0.9367160   17.00
124   53507606 2.6315789   51.8400000   10.00
125   53507606 3.8400000    2.4247324   12.00
126   40130705 4.1666667    9.4946056   12.00
127   40130705 5.6497175    0.2048379   10.00
128   40130705 3.6900369    5.8468852   14.00
129   40130705 2.9520295    3.4775013    4.00
130   40130705 2.8571429   49.7761333    2.00
131   40130705 4.2158516    2.0026382   19.00
132   26753803 4.3478261    6.2402419   12.00
133   26753803 3.7735849    2.0567578   10.00
134   26753803 4.5045045   16.7690250   20.75
135   21403042 4.6948357   10.4112241   42.00
136   21403042 4.4052863   15.6085775   20.75
137   21403042 5.2356021    9.1692887   33.00
138   21403042 5.0505051   16.9883469   23.00
139   21403042 5.4495913   14.3816599   39.00
140   21403042 5.2083333    6.8599093   35.00
141   21403042 5.2356021    6.7173532   28.00
142   10701521 6.4516129   10.2133507   28.00
143   10701521 6.4935065    0.2862860   14.00
> model2 <- nls(R1 ~ ((R1A*1e4*omega^(-0.6))+(0.01*R1B*1.4274*log(1+(1e-11*1.4274*omega)^(-
2))) +(0.04*R1B*1.4274*log(1+(2e-11*1.4274*omega)^(-2)))+0.213), start=list(R1A=7.5,
```

```
R1B=1.25), weights=R1w, control=list(maxiter = 500, tol = 0.001, minFactor = 1e-7, printEval
= TRUE, warnOnly = FALSE))
  It.   1, fac=           1, eval (no.,total): ( 1,  1): new dev = 1077.31
>  temp<-summary(model2,100); print(temp);

Formula: R1 ~ ((R1A * 10000 * omega^(-0.6)) + (0.01 * R1B * 1.4274 * log(1 +
    (1e-11 * 1.4274 * omega)^(-2))) + (0.04 * R1B * 1.4274 *
    log(1 + (2e-11 * 1.4274 * omega)^(-2))) + 0.213)

Parameters:
    Estimate Std. Error t value Pr(>|t|)
R1A  8.66288    0.68146   12.71   <2e-16 ***
R1B  1.29413    0.08243   15.70   <2e-16 ***
---
Signif. codes:  0 '***' 0.001 '**' 0.01 '*' 0.05 '.' 0.1 ' ' 1

Residual standard error: 2.901 on 128 degrees of freedom

Correlation of Parameter Estimates:
    R1A
R1B -0.85

Number of iterations to convergence: 1
Achieved convergence tolerance: 1.619e-09
```

Supplementary material 4: additional exploratory fitting

	Weighed by inverse inter-subject variance	Weighted by number of subjects	Unweighted
Fit A, B to model			
A/10^4	**8.66288 ± 0.68146**	8.74379 ± 0.31379	8.11817 ± 0.40905
B/10^9	**1.29413 ± 0.08243**	1.17456 ± 0.06623	1.34326 ± 0.09147
Correlation of A, B estimates	**-0.85**	-0.83	-0.85
Fit A, B, $R_{1,\infty}$ to model			
A/10^4	5.9457 ± 1.0221	8.40593 ± 0.68768	7.8964 ± 0.7742
B/10^9	2.5546 ± 0.3729	1.42621 ± 0.46029	1.4990 ± 0.4700
$R_{1,\infty}$	-0.3373 ± 0.1591	0.07332 ± 0.25282	0.1285 ± 0.2501
Correlation of A, B estimates	-0.87	-0.94	-0.92
Correlation of A, $R_{1,\infty}$ estimates	+0.77	+0.89	+0.85
Correlation of $R_{1,\infty}$, B estimates	-0.98	-0.99	-0.98
Fit A, B, τ_D to model			
A/10^4	5.86036± 1.07165	8.3975± 0.6934	7.8945± 0.7785
B/10^9	0.54718± 0.06614	0.7105± 0.5042	1.0071± 0.7568
τ_D	6.81912± 1.72191	2.8792± 2.9580	2.1268 ± 2.2481
Correlation of A, B estimates	+0.26	+0.84	+0.79
Correlation of A, τ_D estimates	-0.68	-0.88	-0.84
Correlation of τ_D, B estimates	-0.87	-1.00	-1.00
Fit A, B, k to model			
A/10^4	0.6380± 2.5022	1.0623± 1.6433	3.3827± 5.4989
B/10^9	0.3250± 2.7350	0.3627± 0.9328	1.1064± 0.5231
k	0.4309± 0.2704	0.4618± 0.1052	0.5437± 0.1055
Correlation of A, B estimates	1.00	+0.99	+0.97
Correlation of A, k estimates	1.00	+1.00	+1.00
Correlation of k, B estimates	1.00	+1.00	+0.98
Fit to heuristic			
M	-0.361076± 0.011526	-0.390349± 0.008051	-0.370005± 0.010705
C	0.295561± 0.007289	0.268403± 0.006671	0.282139± 0.008855
Correlation of M, C estimates	-0.26	+0.66	+0.70
*Contribution of studies published ≤1992**	*4%*	*27%*	*38%*

**Figure 3 in the main manuscript suggests that some studies published ≤1992 exhibited higher variability and may therefore be less reliable. This row indicates how ≤1992 studies contribute to the fits under the different weighting schemes.*

These fifteen fits are plotted below. The primary 2-parameter weighted fit to the model is shown in the table and figure in bold black; two secondary 2-parameter fits to the model are shown in the table and figure in blue; nine secondary 3-parameter fits to the model are shown in the table and figure in red; and three secondary 2-parameter fits to the heuristic are shown in the table and figure in green. Note that, in the three-parameter fits, some of the fitted parameters are unphysiological, there are very high correlations between some of the parameters, and some of the errors are very large, so these are probably not useful.

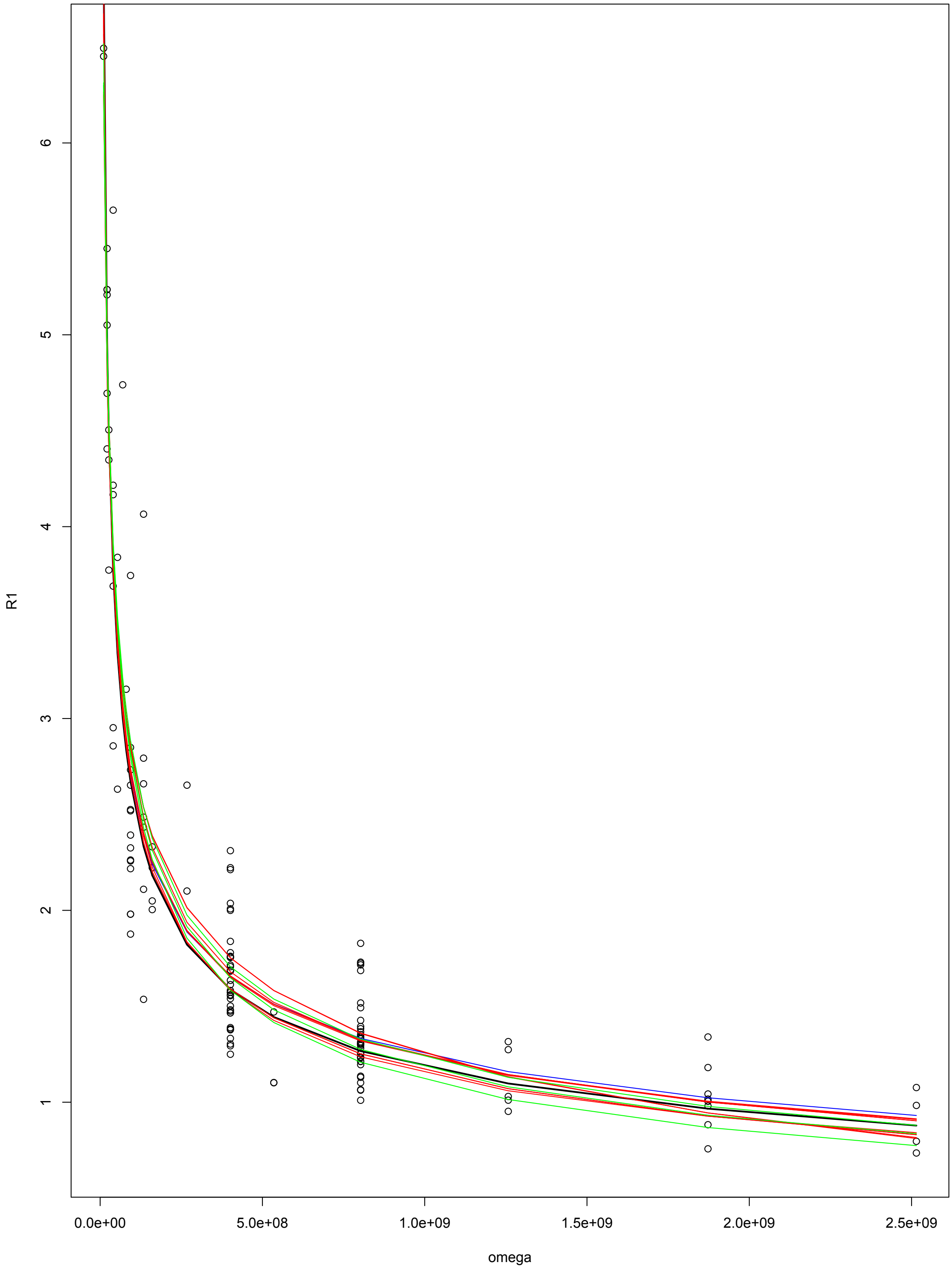
R1
6
5
4
3
2
1
0.0e+00
5.0e+08
1.0e+09
1.5e+09
2.0e+09
2.5e+09
omega

Supplementary material 5: common methods

Class	Definition	Number of studies	Increments median(range)	Median year of publication
Inversion recovery (IR)	An inversion pulse, a TI delay, then any kind of readout. At least two different TI delays are employed.	26	8(2..20)	2004
Saturation recovery (SR)	Saturation, a TS delay, then any kind of readout. At least two different TS delays are employed.	30	2(2..20)	1998
Lock-Locker (LL) (including modified LL)	An inversion pulse, then, during return to equilibrium, there is a series of delays punctuated by transverse magnetisation creation and sampling. At least two samples are taken.	35		2018
Variable flip angle (VFA)	Transverse magnetisation is created, followed by any kind of readout, then a TR delay. At least two different flip angles are employed to create the transverse magnetisation.	115	2(2..5)	2015
"mixed"	Methods using different sequences, solved analytically, or using lookup tables	25		1987
MR fingerprinting (MRF)	Methods using different sequences on a large scale, solved using dictionaries	2		2017
Multiple, unclear and other	Includes studies in which more than one method was employed	11		
total		143		2004

Supplementary material 6: subgroup analyses

Analyses for specific species and fields

species	B_0 (T)	Mean R_1 (s^{-1}) over all studies			Best fit to model Eq.3	rms standard deviation (s^{-1})		Number of studies (number of subjects)
		Weighted by						
		inverse between-subject variance	number of subjects	not		Over all studies	Over ≥1992 studies	
mouse	9.4	0.76	0.92	0.93	0.92	0.11	0.11	3(28)
mouse	7	0.91	0.96	1.00	1.02	0.04	0.04	5(26)
rat	7	1.12	1.03	1.06		0.10	0.10	4(30)
mouse	4.7	1.03	1.01	1.00	1.15	0.07	0.07	3(29)
rat	3	1.62	1.67	1.59	1.33	0.14	0.14	6(50)
man	3	1.24	1.27	1.28		0.14	0.14	28(926)
man	1.5	1.62	1.54	1.65	1.66	0.27	0.16	32(1664)

Does R_1 deviate for subgroups?

There was no evidence R_1 values for subgoups based on species or method deviated systematically from Eq.3. The mean±SD difference between observed R_1 values and the fit to Eq.3 were:

Mouse: (-3±16)%
Rat: (+7±25)%
Man: (-3±15)%
IR: (-1±17)%
SR: (-9±17)%
VFA: (+2±17)%
LL: (+2±14)%

Supplementary material 7: early vs post-1992 studies

The pre/post 1992 distinction in intra-study inter-subject CoV (figure 3, main manuscript) seemed striking enough to justify a post-hoc analysis. This may reflect the relatively primitive technology available in the 1980s together with skills shortages in MR physics during that period. It seems unjustified simply to discard all ≤1992 studies, as these are the main source of the low-field data, and some of the studies do appear to have been carefully performed by highly-skilled teams. The tactic of weighting by the inverse of the inter-subject variance massively diminishes the contribution of the high-variance studies. Thus, while ≤1992 studies constituted 37% of the studies and 26% of the N, they only contributed 4% of the weight.

Supplementary material 8: variance components

R_1 variance component:	Repeatability within-study within-subject (wsws) between-scan	Within-study between-subject (wsbs)	Between-study, different subjects (bsbs), see note
Type of record	Test-retest CoV_{wsws}	Within-study between-subject CoV_{wsbs}	Study R_1 normalised to model (Eq.3)
Number of records: total / >1992	8 / 7	131 / 81	141 / 88
metric	Average $(CoV_{wsws})^2$	Average $(CoV_{wsbs})^2$	$(CoV_{bsbs})^2 = (SD/mean)^2$ of normalised study R_1 value
variance >1992 studies	0.34×10^{-3}	8.8×10^{-3}	26.0×10^{-3}
fractional variance >1992 studies (sum=100%)	0.98%	25.1%	73.9%
√(variance) >1992 studies	1.86%	9.4%	16.1%
variance all studies	0.37×10^{-3}	9.2×10^{-3}	31.5×10^{-3}
fractional variance all studies	0.90%	22.3%	76.8%
√(variance) all studies	1.93%	9.6%	17.8%

Note: in order to eliminate the expected contribution of B_0 to the variance, each study R_1 value was divided by the fitted value for the appropriate B_0 using Eq.3. Alternative estimates of CoV_{bsbs}^2 were obtained by averaging CoV_{bsbs}^2 at each field strength, and by eliminating values from small studies: these yielded very similar values
